# Does Plant Size Influence Leaf Elements in an Arborescent Cycad?

**DOI:** 10.3390/biology7040051

**Published:** 2018-12-13

**Authors:** Thomas E. Marler, Murukesan V. Krishnapillai

**Affiliations:** 1 College of Natural and Applied Sciences, University of Guam, Mangilao, Guam 96923, USA; 2 Cooperative Research and Extension, College of Micronesia-FSM, Yap Campus, Yap 96943, Micronesia; muru@comfsm.fm

**Keywords:** allometry, *Cycas micronesica*, growth rate hypothesis, resorption efficiency, resorption proficiency

## Abstract

Plant size influences the leaf nutrient relations of many species, but no cycad species has been studied in this regard. We used the arborescent *Cycas micronesica* K.D. Hill to quantify leaf nutrient concentrations of trees with stems up to 5.5-m in height to determine if height influenced leaf nutrients. Green leaves were sampled in a karst, alkaline habitat in Rota and a schist, acid habitat in Yap. Additionally, senesced leaves were collected from the trees in Yap. Minerals and metals were quantified in the leaf samples and regressed onto stem height. Green leaf nitrogen, calcium, manganese, and iron decreased linearly with increased stem height. Senesced leaf carbon, iron, and copper decreased and senesced leaf nitrogen increased with stem height. Nitrogen resorption efficiency decreased with stem height. Phosphorus and potassium resorption efficiencies were not influenced by plant size, but were greater than expected based on available published information. The results indicate leaf nutrient concentrations of this cycad species are directly influenced by plant size, and illuminate the need for adding more cycad species to this research agenda. Plant size should be measured and reported in all cycad reports that include measurements of leaf behavior.

## 1. Introduction

The mineral nutrients and other trace elements within an organism are collectively called the ionome [1]. Foliar nutrient concentrations of plants have been studied as a component of the ionome in many horticulture, agronomy, forestry, and ecology disciplines. Plant size is one of the plant traits that influences leaf nutrients, with nutrient concentrations in leaf tissues generally decreasing with increased plant size [2,3,4,5]. Phosphorus (P) concentration in particular tends to decrease in leaves as plant size increases more so than other essential elements.

The influence of plant size on leaf nutrient concentrations of a cycad species has not been directly studied. In fact, plant size was not included in most of the papers reporting cycad leaf nutrient data [6,7,8,9,10]. Two cycad reports that contained leaf nutrient concentrations did include plant size, but the direct influence of plant size on the leaf traits was not discussed in either report [11,12]. Ecophysiological leaf traits of *Dioon sonorense* (De Luca, Sabato, and Vazq.-Torres) Chemnick and T.J. Greg and Salas-Morales were reported along with 14 environmental variables for comparison [13]. The role of ontogeny was among the discussed traits, and leaf nitrogen (N) concentration was greater in seedlings than in juveniles or adults. No other leaf element was included in their analysis. Therefore, no studies to date have reported how cycad plant ontogeny or size influences a broad spectrum of leaf minerals and metals. This limits our understanding about how to appropriately sample green cycad leaf tissues to determine plant health status or how to unambiguously determine covariation of leaf elements among cycad species.

Our objective was to use *Cycas micronesica* K.D. Hill as a representative cycad species to determine the influence of plant size on leaf elemental concentrations in young fully expanded green leaves and senesced leaves. We used height of the pachycaulous stem as the metric for plant size. We selected a habitat in alkaline coralline soils and a habitat in acid schist soils to provide a contrast in soil chemistry. We hypothesized that *C. micronesica* plants (1) would exhibit greater concentrations of elements in leaves of small plants in conformity with the literature, (2) elemental patterns would differ between the two contrasting habitats, and (3) since cycads have access to atmospheric N via cyanobacteria endosymbionts [14], N concentrations and resorption characteristics would be less responsive to variation in plant size and soil chemistry than other essential nutrients.

## 2. Materials and Methods

### 2.1. Locations

We studied two in situ *C. micronesica* populations in Rota (14°07′36″ N, 145°07′42″ E) and Yap (9°32′16″ N, 138°06′38″ E). The Rota soil series was formed in sediment overlying limestone (loamy, oxidic, nonacid, isohyperthermic Lithic Ustorthents) [15]. The chemical traits of this calcareous soil are characteristic of most karst and sand habitats that support high density *C. micronesica* populations in Guam, Palau, and Rota. Sampling dates were 11–14 October 2007. At this time, there were no other habitats on Guam or Rota where *C. micronesica* plants were healthy and free from non-native invasive specialist insect pests. The Yap soil series was formed in residuum derived from green, chlorite, and talc schist (clayey-skeletal, mixed, isohyperthermic Lithic Tropudalfs) [16]. Sampling dates were 12–15 October 2016. To date, all *C. micronesica* habitats in Yap have not been invaded by non-native insect pests and the plants are not facing any known threats.

### 2.2. Field Methods

**Figure 1:**
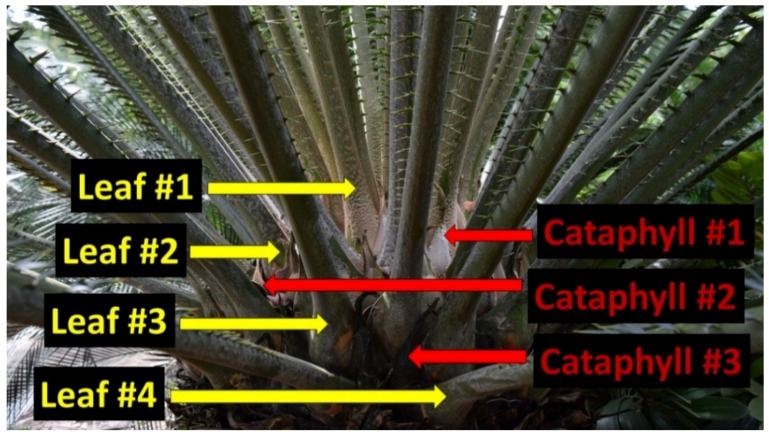
Production of cataphylls (red arrows) unambiguously demarcates the sequential separation of chronological leaf flushes (yellow arrows) exhibiting different historical leaf construction dates for arborescent cycad plants. The youngest leaves (#1) are apical to the youngest cataphylls (#1). This cycad plant contains leaves of four distinct age categories.

Our methods followed established sampling protocols for leaf tissue analyses. The Rota leaf sample collections were designed to quantify essential nutrient concentrations in the youngest fully expanded green leaves. The massive apex of a cycad stem is protected by modified leaves called cataphylls [14]. A newly added leaf flush emerges from the center of these cataphylls, pushes them outward, then they remain within the retained leaf crown and serve as a permanent demarcation between successive leaf flushing events (Figure 1). Therefore, identifying the leaves from the most recent flush on a previously unvisited plant is an unambiguous endeavor. We collected leaflets from every leaf within the youngest leaf cohort.

The Yap leaf sample collections followed these same protocols but also included senescent leaf samples for quantifying leaf nutrient resorption behaviors [17,18]. We added carbon (C) to the list of elements for the senescent leaves. We harvested leaflet samples from healthy green leaves and from senescent leaves on the same plants. Senescent leaves were restricted to leaves still attached to the plants with fully senescent leaflets but rachis and petioles that were not fully senescent. This ensured that we sampled the most recently senesced leaflet tissue to minimize mineral leaching losses from the suspended litter. We collected leaflets from every senesced leaf that met these criteria. For green and senesced leaves, entire leaflets were collected from the apex, midpoint, and base of each leaf rachis and comingled into one sample. The plants were selected to provide a full range in plant height from each habitat but to maintain similar incident light for each plant. Our efforts to ensure similar incident light among all sampled plants was to safeguard the methods against confounding the leaf element concentrations with heterogeneous incident light among the height categories. This required the location of seedlings and juveniles that were not directly beneath mature *C. micronesica* trees. In total, there were 42 plants from Rota and 36 plants from Yap, and the tallest height was 554 cm in Rota and 419 cm in Yap.

We also collected samples in the surface 15-cm layer of soil at half the radius of the dripline of each plant. For monopodial cycad trees, this is comparable to half the length of the longest leaves. Four soil subsamples were collected for each plant, one in each cardinal direction. The four subsamples for each plant were homogenized to provide a single soil sample for each plant. This approach enabled an accurate measure of soil chemistry following plant–soilplant–soil feedback changes within the footprint of each plant, since *C. micronesica* is a local driver of soil nutrient change [19]. Therefore, the data provide more accurate assessments of the edaphic conditions of the experimental plants rather than the general traits of the soils in the habitats. During the field work, the soil samples were numbered sequentially in the order that each plant was added to the accumulating data set. These protocols enabled the sampling from a total of 168 sites among 42 trees in Rota and 144 sites among 36 trees in Yap.

We used a 0.75-m line quantum sensor (EMS-7, PP Systems, Amesbury, MA, USA) to quantify the incident light for each sampled plant. The percentage photosynthetically active radiation (PAR) transmission at the location of each sample was quantified by placing one sensor in the direct incident solar beam and a second sensor beneath the emergent forest canopy where each *C. micronesica* leaf was positioned. Measurements were conducted from 1100 h to 1300 h. The percent transmission of ambient light was calculated from the difference in PAR.

### 2.3. Laboratory Methods

The leaf tissue was dried at 75 °C for 48 h and milled to pass through a 20-mesh screen. In order to reduce the number of digestions to a manageable level, the heights of every sampled plant were ranked in order, then the leaf samples of each group of three plants (starting with the shortest and proceeding to the tallest) were combined to create 14 height categories for Rota and 12 height categories for Yap. Total N and C were determined by dry combustion (FLASH EA1112 CHN Analyzer, Thermo Fisher, Waltham, MA, USA) [20]. Samples were also digested by a microwave system with nitric acid and peroxide, then phosphorus (P), potassium (K), calcium (Ca), magnesium (Mg), iron (Fe), manganese (Mn), zinc (Zn), and copper (Cu) were quantified by inductively coupled plasma optical emission spectroscopy (Spectro Genesis; SPECTRO Analytical Instruments, Kleve, Germany) [21].

Groups of soil samples were combined to reduce the analytical methods to a manageable number of replications. We employed a random approach by standardizing both sites to have six total soil samples by combining soils from seven trees from Rota or six trees from Yap into each sample. The groups of samples were in accordance with the order in which they were collected in the field. Total N of soil samples was determined by dry combustion. Minerals and metals were extracted by digesting in diethylenetriaminepentaacetic acid, then quantified by inductively coupled plasma optical emission spectroscopy.

### 2.4. Derived Variables and Statistics

The PAR data were subjected to analysis of variance (ANOVA) to determine if incident PAR varied among the height categories (12 for Yap and 14 for Rota) with three replications (SAS 9.3; SAS Institute, Cary, Indiana). Nutrient resorption efficiency from Yap trees was calculated as ((nutrient_green_ − nutrient_senesced_)/nutrient_green_) × 100. Nutrient resorption proficiency was taken as the concentration of senesced leaves in accordance with Killingbeck [17]. All response variables were fitted to linear and quadratic models with tree height as the independent variable using the Proc GLM procedure in SAS. Each of the 14 Rota or 12 Yap independent height data points were calculated as the mean for each of the three plants that were combined into each of the single tissue samples.

## 3. Results

The Rota alkaline, karst soils contained greater N, P, K, Ca, and Mn than the Yap acid, schist soils (Table 1). In contrast, the Yap soils contained greater Fe and Cu than the Rota soils. Concentrations of Mg and Zn were similar in the two soils.

Our approach to ensure homogeneous incident light among the height categories was successful, as the PAR did not differ among the Rota height categories (*p* = 0.2701) or Yap height categories (*p* = 0.6034). Percent PAR transmission was 14% ± 3% for Rota and 6% ± 1% for Yap.

**Figure 2:**
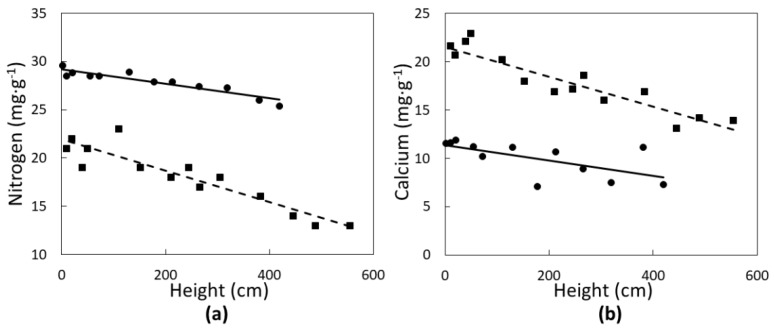
The influence of *Cycas micronesica* tree height on (**a**) nitrogen and (**b**) calcium green leaf concentration in Rota (squares and dashed line) and Yap (circles and solid line).

None of the leaf elements followed a quadratic relationship with tree height. Two of the macronutrients were significantly influenced in a linear fashion by *C. micronesica* plant height. Nitrogen concentrations of green leaves (Figure 2a) declined with increased plant height in Rota (*r*^2^ = 0.83; *p* < 0.0001) and Yap (*r*^2^ = 0.86; *p* < 0.0001). Calcium concentrations of green leaves (Figure 2b) also declined linearly with increased plant height in Rota (*r*^2^ = 0.74; *p* < 0.0001) and Yap (*r*^2^ = 0.43; *p* = 0.0018). Rota plants exhibited a greater range in N and Ca than Yap plants.

**Figure 3:**
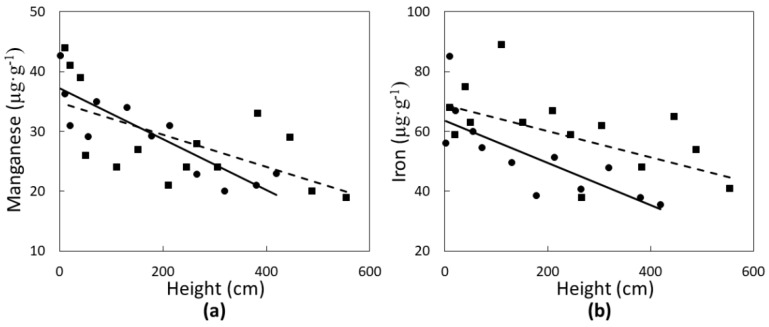
The influence of *Cycas micronesica* tree height on (**a**) manganese and (**b**) iron green leaf concentration in Rota (squares and dashed line) and Yap (circles and solid line).

Two micronutrients were significantly influenced in a linear fashion by *C. micronesica* plant height. Manganese concentrations of green leaves (Figure 3a) declined with increased plant height in Rota (*r*^2^ = 0.38; *p* = 0.0186) and Yap (*r*^2^ = 0.70; *p* = 0.0256). Similarly, iron concentrations of green leaves (Figure 3b) also declined linearly with increased plant height in Rota (*r*^2^ = 0.29; *p* = 0.0413) and Yap (*r*^2^ = 0.55; *p* = 0.0061). The range of Mn and Fe was greater than that of N or Ca, and micronutrient concentration of the green tissue was more similar for the two locations than for the macronutrients.

No other macro- or micronutrients of green leaves were influenced by *C. micronesica* plant height. However, nutrient resorption proficiency decreased with plant height in the Yap soils for C, Fe, and Cu (Table 2). In contrast, N resorption proficiency significantly increased with increased plant height. The N resorption efficiency (NRE) decreased with plant height in the Yap soils.

Green leaf response variables that did not exhibit a dependence on plant height included the concentrations of P, K, Mg, Cu, and Zn (Table 3). Senesced leaf response variables also included those that were not dependent on plant height. Resorption proficiency of P, K, Ca, Mg, Mn, and Zn and resorption efficiency of P (PRE) and K (KRE) were unaffected by plant height in Yap soils (Table 4).

## 4. Discussion

We have shown that plant size influenced leaf element behavior in the arborescent cycad *C. micronesica*. This is the first cycad report covering a broad range of leaf elements that included a dedicated look at the influence of plant size. That some of the leaf elements were influenced by plant size was expected, given the role of plant size in many issues of plant life history strategy [22]. The unfortunate consequence of these outcomes is that leaf element results reported in most previous cycad papers [6,7,8,9,10] are ambiguous and cannot be repeated due to the failure to measure and report plant size.

The results confirmed our first hypothesis in both habitats for green leaf N, Ca, Mn, and Fe concentrations and for senesced leaf C, N, Fe, and Cu in Yap soils. Senesced leaf N was positively dependent on plant height, but every other significant response variable was negatively dependent on plant height. The lack of P dependence on plant height was unexpected because P is one of the most consistent elements to conform to this relationship of a decrease with increased plant size [1].

The results also confirmed our second hypothesis. Two of the environmental factors that contrasted our two habitats were soil chemistry (alkaline karst in Rota versus acid schist in Yap) and incident light (incident PAR of Rota more than double that of Yap). The habitat with higher soil Ca concentration contained trees with greater leaf Ca. The remainder of the habitat differences in leaf nutrient concentrations were not correlated with differences in soil nutrient concentrations. The confirmation of this hypothesis reveals that the reporting of cycad leaf nutrient data cannot be fully interpreted if environmental factors are not measured and reported along with the leaf data.

The results rejected our third hypothesis. Indeed, leaf N traits emerged as a suite of traits that were highly dependent on plant size. Absolute N concentrations of green leaf tissue and NRE were among the response variables that significantly declined with plant size, and N concentration of senesced leaf tissue was the only response variable that increased with plant size.

### 4.1. Cycads

The only other cycad paper to include variation in leaf nutrients with variation in plant size was a general comparison of seedlings, juveniles, and adults of *Dioon sonorense* [13]. Leaf N concentration was the only nutrient that was measured, and it was greatest in seedling leaves. This study illuminates two issues of relevance to our results. First, the *D. sonorense* study involved three discrete size categories rather than a continuous independent height variable to portray plant size. Therefore, ours is the first cycad study to quantify plant size in a manner that enabled an estimate of causation through regression analysis. Second, leaves from the *D. sonorense* seedling category exhibited greater N concentration than leaves from the larger plants, but the juvenile and adult categories exhibited similar leaf N concentrations. This indicated a nonlinear relationship between leaf nitrogen and plant height for *D. sonorense*, which is in contrast to our linear dependence of leaf nitrogen on *C. micronesica* plant height. The height limits for *D. sonorense* were about 2-m, yet the linear decline in leaf N for *C. micronesica* extended to our greatest height of 5.5-m. These two contrasting studies suggest that the influence of cycad plant size on leaf N does not adhere to a canonical pattern among cycad taxa.

The use of tree height is an adequate measure of plant size or allometry for all of the arborescent cycad species. The ultimate height of the pachycaulous stem can be unambiguously identified by the presence of the unique cataphylls. However, many cycad species are not arborescent and develop subterranean stems [14]. Plant height has no relationship to overall plant size for these species. A different plant size metric such as total number of leaves, number of stem apices, or total diameter of the assemblage of stem apices may prove to be appropriate for quantifying allometric relationships for these cycad species.

The scarcity of published cycad leaf element data limits our understanding of covariation among cycad species, organs, and constituents of the ionome. Moreover, the failures to adequately measure and report traits that partially control leaf elements in previous cycad publications magnifies these limitations. Indeed, control over nutrient relations of plants is multifactorial, complicating our ability to understand how plants acquire and manage essential nutrients. The marked variation we have reported in *C. micronesica* leaf element concentrations among height categories reinforces the need to quantify and report some metric of plant size when studying cycad leaf traits. The observation that *C. micronesica* leaf element concentrations are dissimilar between two habitats further reinforces the need to report cycad habitat traits such as soil nutrient data. Omitting these covarying traits from studies of cycad leaf chemistry does not allow a valid discussion about the individual elements that comprise the ionome of cycads.

### 4.2. The Elements

The relative order of elements that we quantified in most plant tissue is generally N > K > Ca > Mg > P > Fe > Mn > Zn > Cu [23]. Our Yap data conformed to this general ranking. In contrast, Ca and Zn did not conform to the general ranking for Rota, as both elements increased one place in the relative ranking of the elements.

*Cycas micronesica* leaf Ca appears to be highly plastic and responsive to soil chemistry and plant size. Plant soil feedback phenomena of this species also increases soil Ca concentration in the vicinity of its roots in acid soils with relatively low ambient Ca concentrations [19]. The inclusion of Ca in continuing cycad leaf nutrient research may provide interesting distinctions about the cycad ionome.

The influence of plant size on leaf nutrients has been reported for various angiosperms and gymnosperms, and summarized to reveal that nutrient concentrations in green leaf tissues generally decrease with increased plant size [2,3,4,5]. However, there are exceptions that do not conform to this general rule. For some tree species, green leaf N concentration increased with age and size [24,25] and for other species green leaf N concentration was not influenced by plant size [25,26,27]. Our results provide a beginning to more fully understand the role of plant size on various elements in cycad leaves. The inclusion of more species in similar studies is needed to determine if the elements that exhibited a dependence on *C. micronesica* plant size behave similarly among other cycad taxa.

### 4.3. Resorption

Nutrient resorption is a critical behavior among plants enabling a recycling of limiting elements prior to organ senescence [17,18]. This behavior affects plant processes by reducing the dependence on the rhizosphere to satisfy ongoing needs of mineral nutrition. Additionally, the behavior affects biogeochemical cycling by increasing elemental residence time within the plant body and reducing the minerals that are deposited into the litter layer when senescent organs are abscised from a plant. Our NRE was 24%, which was substantially lower than the global average of 50 to 60% [17,28,29,30,31]. Our PRE was 66%, which was greater than the global average of 50 to 60% [17,28,29,30,31]. The KRE was 93% for these *C. micronesica* trees, which was substantially greater than the global average of 70% [17,28,29,30,31]. Relatively low NRE and relatively high PRE and KRE were also reported for *Cycas nitida* K.D. Hill & A. Lindstr. [7]. *Cycas wadei* Merrill [12] exhibited low NRE but expected PRE and KRE. Based on our studies on these three western Pacific Cycas species, cycads are relatively inefficient in N resorption but highly efficient in P and K resorption during leaf senescence.

Plant height directly influenced *C. micronesica* leaf NRE, which decreased with plant size. The smallest plants were not only able to retranslocate more of the N that was available in the green leaves, they were also able to reduce the absolute N content to a lower limit in their senesced leaves than the largest plants. This is in contrast with earlier reports indicating greater NRE for larger plants than for smaller plants [25,32,33].

Killingbeck [17] introduced the term resorption proficiency to describe the basal levels to which each nutrient was reduced in senesced leaves. Among woody perennials, a N resorption proficiency of <7 mg·g^−1^ and a phosphorus resorption proficiency of <0.5 mg·g^−1^ indicates complete withdrawal. Our resorption proficiencies did not approach these lower limits. The allometric influences on senesced leaf N are of great importance in understanding the impacts that cycad plants exert on landscape level processes, especially because N is the primary growth-limiting nutrient in many soils throughout the world. After C, hydrogen, and oxygen, N is the next most common plant element, and as a result this single nutrient often limits biomass production [34]. All cycad plants associate with N-fixing cyanobacteria symbionts, so they are able to access atmospheric N and are not reliant on mineral N pools from the rhizosphere for their own growth and productivity [14]. This N is temporarily sequestered within the plants, then is ultimately released from senescent cycad organ litter to the metacommunity where the N becomes mineralized and exerts a strong influence on the food web. Indeed, N availability directly affects growth of primary producers in a landscape, but it also exerts cascading effects on food web functioning among many trophic levels [35]. Our results indicate that *C. micronesica* plant size exerts a direct influence on this process. A senescing leaf from a small plant contributes less biomass with greater N concentrations, and a senescing leaf from a large plant contributes substantial biomass with lower N concentrations.

Why were PRE and KRE greater than expected and NRE lower than expected? We suggest two complementary plant behaviors enabled this outcome. First, the retranslocation of leaf resources during senescence is linked to the sink strength of the subtending stems. The pachycaulous cycad stem is a large structure relative to whole plant size, and is comprised primarily of living parenchyma tissue [14,36]. We suggest the cycad stem exerts much greater sink strength for mineral nutrients than stems in most woody angiosperm and gymnosperm species that have been studied. The result is a PRE and KRE which is much greater than the global means of 50% to 70% [17,28,29,30,31]. This stem pool of minerals is then readily available for deployment during any ephemeral sink activity in plant modules that are external to the stem, such as the construction of a leaf or reproductive structure on the stem apex. Second, assessments of a range in plant species have shown that legumes which associate with N-fixing root symbionts express reduced NRE in comparison to other species [37,38]. In conformity with this literature on legumes, the association with a N-fixing endosymbiont allows the cycad plant to access atmospheric N in addition to soil-derived mineral N. A small plant does not possess a large stem containing copious stored nutrients, nor is it supported by a large assemblage of coralloid roots to house cyanobacteria. In contrast, a large plant is comprised of a relatively large stem structure storing copious mineral resources. This N pool appears to dial down the need for N resorption during leaf senescence in large cycad plants.

We recently reported that microsite soil changes occur beneath mature *C. micronesica* trees such that N and Ca were generally increased while P and K were generally decreased when compared to soils away from *C. micronesica* trees [19]. Our results herein provide relevant observations that may explain how this arborescent cycad generates these soil changes over time. Most or all of the green leaf N and Ca was ultimately contained in the senesced leaf litter, but a small percentage of the green leaf P and K remained in the leaves until senescence. The soil P and K resources that are absorbed by the plant largely persist inside the plant body despite disposal of senescing leaves over time.

### 4.4. The Habitats

Our inclusion of plants from two disjunct habitats revealed contrasting leaf element concentrations for several nutrients. These results suggest that elemental concentrations and stoichiometry of *C. micronesica* leaves are not homeostatic among contrasting habitats. However, the factors that determine site-to-site variation in leaf elements are complex.

Soil nutrition status is one site factor that is closely related to the concentration of leaf nutrients [39,40], a concept that justifies fertilization in managed systems. Moreover, nutrient resorption [38,41,42] and stoichiometry [43,44,45] are also responsive to fertilization and soil nutrient levels. With the exception of Ca, our habitat differences in concentration of leaf elements did not follow the same direction as the differences in concentration of corresponding soil elements. This phenomenon has been reported for other cycad species. For example, a 66-fold difference in soil K did not influence leaf K differences for *C. nitida* trees growing in four Philippine habitats [7]. Similarly, four cycad species growing in two ex situ gardens with contrasting soil nutrients exhibited idiosyncratic relationships of leaf element differences and soil element differences [8]. Clearly, differences in soil nutrient status among habitats do not fully control the behavior of cycad plants with regard to differences in leaf nutrient status.

A second factor that contrasted our Rota and Yap habitats was incident light at the leaf level. The Rota plants experienced 2.3-fold higher levels of PAR transmission than the Yap plants. The mean estimated photosynthetically photon flux for the Rota plants was 294 µmol·m^−2^·s^−1^, but only 126 µmol·m^−2^·s^−1^ for the Yap plants. These two islands are positioned in the most active tropical cyclone basin worldwide [46], and the chronic storms constrain the height of the emergent canopy of the forests through repeated canopy damage. However, the low latitude of Yap causes the frequency of tropical cyclones to be comparatively less, so the forest emergent canopy is taller and exhibits fewer gaps than that of Rota. Numerous angiosperm and coniferous species have exhibited greater leaf N concentrations in shade than in sun conditions [47,48,49,50,51,52,53]. The influence of incident light on leaf nutrients is not known for any cycad species, illuminating the need to study this phenomenon. Regardless, this disparity in light level between the Rota and Yap habitats may explain our contrasting leaf element results.

A third possible explanation for differences in our Rota and Yap concentrations is differences in pressures for trait selection between the two disjunct populations. The dates of the initial colonizing events for each island within the *C. micronesica* indigenous range are unknown, so the amount of time that differential trait selection may have created contemporary differences among the populations is also unknown. The intrinsic needs for leaf nutrients may be under selection in each environment.

### 4.5. Complications

Two aspects of the relevant literature should be considered during ongoing research into how plant size affects cycad leaf chemistry. First, a large spatiotemporal heterogeneity in light is a defining characteristic of the natural environment. Generally, seedlings are more shaded than mature plants because incident light increases with increasing vertical stratification in a forest. Since the incident light level of sampled leaves can change leaf nutrient concentrations [32], stoichiometry [54], and resorption [32], there is no way to remove the influence of heterogeneous incident light level of each size category from the results. Therefore, reported differences in seedling leaf nitrogen versus mature plant leaf nitrogen [13], may be due to differences in incident light rather than differences in plant size. Our stringent control of incident light for each plant regardless of size removed this ambiguity from our methods, leaving plant height as the only known factor controlling the quantified variations in leaf nutrient levels. Second, many of the papers that emerge in literature searches compared stand age, not plant age per se [33,55,56,57,58]. The results in these studies are complicated by the maturing plant–soil feedback changes that co-occur with the increase in plant size. The early years of these studies reported small plant leaf chemistry, but the results were complicated by the fact that the anthropogenic disturbances prior to stand establishment disrupted natural biogeochemical cycling. The later years of these studies reported large plant leaf chemistry, but the trees in these later years also benefitted from years of feedback in soil recovery following the stand establishment. This maturing of plant–soil feedback with stand age undoubtedly exerts its own strong influence on plant leaf chemistry independent of plant size. Therefore, the changes in soil carbon and mineral availability during stand ontogeny obscure the intrinsic role of plant size. Our experimental units were residing in closed canopy forests where the emergent canopy of sympatric plants was a homogeneous age, so we were directly measuring plant size effects on leaf nutrition.

Large-scale comparisons of cycad leaf nutrients among phylogenetic or functional groups are complicated by these findings. If PAR directly affects cycad leaf chemistry, then incident light needs to be included in experimental design by either controlling for it (ensure each sampled leaf experiences similar PAR) or quantifying and communicating it (measure and report PAR). Similarly, if site-to-site variations in rhizosphere chemistry directly affect cycad leaf chemistry, this needs to be included in experimental design by either controlling for it (use a homogeneous substrate for all experimental units) or quantifying and communicating it (report chemistry of soils collected directly adjacent to each plant). Ongoing attempts to address the paucity of reported cycad data should be conducted with experimental methods that clarify these ambiguities.

## 5. Conclusions

Our approach has confirmed an influence of *C. micronesica* plant size on leaf nutrient relations. This is the first study on any cycad species where the role of plant size on leaf chemistry was directly studied. The two contrasting habitats also exhibited differences in leaf nutrients. Our results illuminate the continuing need to identify all plant and environmental factors that influence leaf chemistry such that ongoing cycad research can become more accurate by demanding appropriate experimental protocols. The cascading effects of plant size and rhizosphere chemistry need to be explicitly incorporated into all phylogenetic or ecotypic research programs that include the study of leaf traits in cycad species.

## Figures and Tables

**Table 1:** Soil reaction and mineral and metal concentrations of Rota karst soils and Yap Schist soils in high density *Cycas micronesica* populations. Mean ± standard error, *n* = 6.

Soil Trait	Rota	Yap
pH	7.61 ± 0.05	5.95 ± 0.18
Nitrogen (mg·g^−1^)	14.47 ± 0.78	5.22 ± 0.79
Phosphorus (µg·g^−1^)	88.11 ± 22.14	12.53 ± 1.09
Potassium (µg·g^−1^)	240.24 ± 72.78	99.50 ± 11.85
Calcium (mg·g^−1^)	13.84 ± 0.49	2.03 ± 0.36
Magnesium (mg·g^−1^)	1.04 ± 0.12	1.40 ± 0.17
Manganese (µg·g^−1^)	71.28 ± 11.17	14.15 ± 2.22
Iron (µg·g^−1^)	37.33 ± 8.59	328.67 ± 59.47
Copper (µg·g^−1^)	1.81 ± 0.48	3.87 ± 0.44
Zinc (µg·g^−1^)	10.08 ± 2.72	7.80 ± 1.49

**Table 2:** Linear regression parameters of senesced leaf element response variables with Yap *Cycas micronesica* tree height as the independent variable. Variable = *a* + *b* × height.

Response Variable	*a*	*b*	Regression Coefficient	*p*
Carbon (mg·g^−1^)	524.65	−0.06	0.58	0.0041
Nitrogen (mg·g^−1^)	21.04	0.002	0.46	0.0150
Iron (µg·g^−1^)	198.07	−0.45	0.48	0.0124
Copper (µg·g^−1^)	4.83	−0.01	0.67	0.0011
Nitrogen resorption efficiency (%)	28.10	−0.03	0.88	<0.0001

**Table 3:** Green leaf elemental response variables that were not influenced by *Cycas micronesica* tree height in Rota karst soils or Yap schist soils.

Response Variable	Mean	Minimum	Maximum
	Rota		
Phosphorus (mg·g^−1^)	1.46	1.19	1.68
Potassium (mg·g^−1^)	11.95	6.91	18.42
Magnesium (mg·g^−1^)	6.39	4.48	8.17
Copper (µg·g^−1^)	12.36	6.45	17.93
Zinc (µg·g^−1^)	46.43	24.38	70.22
	Yap		
Phosphorus (mg·g^−1^)	2.27	1.73	2.67
Potassium (mg·g^−1^)	19.06	15.20	23.05
Magnesium (mg·g^−1^)	2.44	1.79	2.90
Copper (µg·g^−1^)	4.19	2.39	7.42
Zinc (µg·g^−1^)	20.8	15.21	37.11

**Table 4:** Senesced leaf elemental response variables that were not influenced by *Cycas micronesica* tree height in Yap schist soils.

Response Variable	Mean	Minimum	Maximum
Phosphorus (mg·g^−1^)	0.74	0.46	0.89
Potassium (mg·g^−1^)	1.38	0.99	1.91
Calcium (mg·g^−1^)	9.41	4.17	15.12
Magnesium (mg·g^−1^)	4.76	3.39	6.52
Manganese (µg·g^−1^)	51.91	24.46	86.12
Zinc (µg·g^−1^)	15.34	4.48	31.21
Phosphorus resorption efficiency (%)	66.38	55.04	82.46
Potassium resorption efficiency (%)	92.67	89.91	95.67
